# Word and Mystery: The Acoustics of Cultural Transmission During the Protestant Reformation

**DOI:** 10.3389/fpsyg.2021.564542

**Published:** 2021-03-02

**Authors:** Braxton Boren

**Affiliations:** Audio Technology Program, Department of Performing Arts, American University, Washington, DC, United States

**Keywords:** room acoustics, archeoacoustics, psychoacoustics, worship spaces, liturgical music, protestant reformation

## Abstract

To a first-order approximation we can place most worship services on a continuum between clarity and mystery, depending on the setting and content of the service. This liturgical space can be thought of as a combination of the physical acoustics of the worship space and the qualities of the sound created during the worship service. A very clear acoustic channel emphasizes semantic content, especially speech intelligibility. An immersive, reverberant acoustic emphasizes mystery and music. One of the chief challenges in acoustical design is the fact that both clarity and immersion are subjectively preferred by audiences, yet these two goals are almost mutually exclusive of one another. The movement along this continuum in liturgical space can also be seen in the religious contexts for many of the worship spaces constructed in the West in the last two millennia. In the case of religious ceremony, a free field acoustic environment provides more clarity and precision in the spoken word received from God and given to the congregation. Yet a diffuse field environment provides an embodied, otherworldly sense of the supernatural: the mystery of the faith received which cannot merely be put into words. This tension is perceptible in many of the religious controversies in the West during this time period. This article examines the history of the spaces used by early Western Catholic Christians as well as those of the traditions—Lutheran and Calvinist—that left the Catholic faith during the 16th century Reformation. By considering the stated goals of these traditions alongside the architectural and liturgical innovations they created, it can be seen that emergent liturgical spaces mirror the assumptions of their respective traditions regarding the proper balance between semantic and aesthetic communication during the worship service. The Reformed faiths' emphasis on the power of the Word is reflected in the liturgical space of their services, while the Catholic faith gave greater priority to the role of Mystery, in their liturgical space as well as their explicit theology. Once constructed, these spaces also aid the cultural transmission of the sung or spoken liturgy of each tradition to future generations.

## 1. Introduction

In the world of music, it is not completely true that “the medium is the message,” yet there is a complex interplay between many different media that account for the final “product” heard by the composer, a period audience, or a listener today. It is common in the analysis of modern music to consider the effects and technologies used to render a song as significant as the traditional sheet music on which it is composed (Katz, 2010). It is certainly true that many aspects of period practice have today been derived by rigorous musicological scholarship, enriching our understanding of the types of instruments and ensembles that would have been used in historical performances of well-known pieces. This is a scientific endeavor which has done much to enrich our understanding of individual instruments and their effect on the final output of the entire ensemble. This necessary progress is still insufficient to develop a wholistic understanding of music as it was received by listeners in the past.

Just as an analysis of a modern pop record would be superficial without considering the reverb or final EQ on the mix, so too a consideration of historical music or a historical speech or sermon is not complete without considering the reverberation and frequency spectrum of the space in which the piece was written, as well as other factors affecting the subjective experience of a listener in the crowd. Such an approach was once impractical because reconstructing a concert hall is much more time-intensive than reconstructing a period flute or viol. But in the past decade greater availability and quality of computational acoustic simulation methods have given us a window into how historical spaces would have sounded in the past (Katz and Wetherill, 2005; Boren and Longair, 2011; Postma and Katz, 2015; Boren, 2019).

This article examines the history of room acoustics and Christian theology in the West, from the first century until the Protestant Reformation in the 16th century. In the context of religious music and liturgy particularly—perhaps more so than instrument design or ensemble size—the final link in the chain of musical production (the room) is intimately connected to the religious context of the acoustic performance spaces, many of which were constructed primarily as worship spaces rather than concert halls. To show this relationship, the article first constructs a new dimension of liturgical space, which constitutes both the acoustic space as well as the clarity of liturgical content in the physical space. Next it examines the history of Western Christianity and its roots in Judaism during the first century, followed by some history of worship spaces under Roman Catholicism and the Protestant Reformation. In each of these cases, different religious criteria were emphasized by different traditions, and these emphases can also be seen in the acoustics of the spaces used by each tradition.

## 2. Background

### 2.1. Room Acoustics

The history of room acoustics may be well-known to many of the readers, but given the interdisciplinary focus of this special issue, some context is given here for those unfamiliar with the basics of the field. The scientific study of the transmission of sound in the West dates back to the ancient Greeks, but the complex behavior of sound in rooms was not really understood until the early 20th century (Lindsay, 1966). For this reason, many buildings were constructed with only hazy intuitions of how their architecture would affect sound transmission once completed. Many sound-critical spaces, such as performance halls, were essentially copies of previous spaces that were known to have satisfactory acoustics. Those architects who attempted to create something new experienced something like Charles Garnier, who described his creation of the Opera Garnier “like the acrobat who closes his eyes and clings to the ropes of an ascending balloon” (Beranek, 2004).

The modern quantitative understanding of room acoustics began with Wallace Sabine, who had been asked to examine the new Fogg Art Museum's lecture theater, which was found to have terrible acoustics for the intelligibility of the voice. Sabine did extensive experiments in the space and decided to quantify its acoustic response by measuring its reverberation time, which he defined as the amount of time necessary for a sound to drop to inaudibility (Katz and Wetherill, 2005). Based on his experiments changing the material absorption present in the room, Sabine eventually produced an equation that quantified reverberation time *T* in terms of room volume *V* and acoustic absorption *A*. If all units are in SI form, Sabine's equation can be expressed as

(1) $$\begin{array}{r} T=0.16\frac{V}{A} \end{array}$$

Sabine's formulation for reverberation time has become the foundational parameter on which the rest of the field has been constructed (Cirillo and Martellotta, 2005). While many more in-depth perceptual parameters are used to quantify a room's acoustic response, mid-frequency reverberation time can still be used as a first-order approximation of the major continuum spanning the field of room acoustics, from rooms with nearly no reverberation to those with a very long reverberation time. This general phenomenon was noticed by Hope Bagenal, another founding voice in the field, who claimed that all modern auditoria had their roots in either the acoustics of “the open air” or the acoustics “the cave” (Bagenal, 1951).

At one end of this continuum we can label the non-reverberant “open air” case (*T*≈0) as an acoustic *free field*, an ideal mathematical construction in which sound radiates from a source with no reflected sound. Free-field models are useful for theoretical calculations about an individual sound wave, but they are only an approximation of reality for special cases like anechoic chambers or outdoor conditions in which most incident sound quickly escapes or is absorbed.

**Figure 1:**
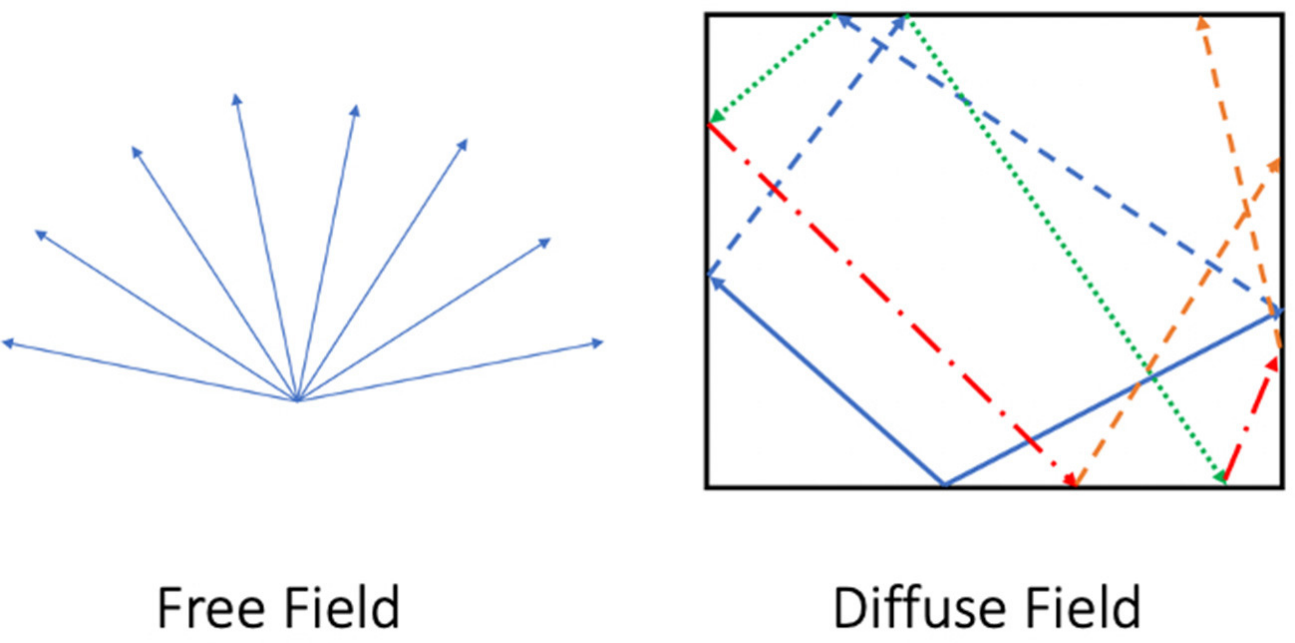
Sound propagation in a free field vs. a diffuse field.

The opposite case of rooms with a very long reverberation time (“the cave”) can be labeled generally as an acoustic *diffuse field*. This is defined as an environment with so many reflections that we cannot treat them individually and therefore must categorize them statistically. Figure 1 shows the difference in sound propagation between free and diffuse field sound environments.

**Figure 2:**
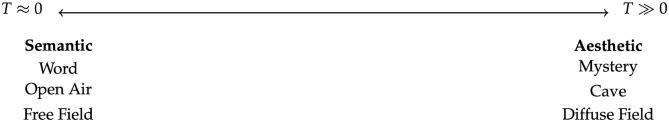
Basic continuum of Liturgical space, from the extremes of semantic to aesthetic.

Bagenal (1951) asks us to consider the fact that though our auditory system largely evolved outdoors in a more or less free field environment, at one point our neolithic ancestors stumbled into a cave with reflective stone walls enclosing them. Whereas, outside they could precisely pinpoint source locations, in the cave localization was more difficult, yet the sound of their voice was amplified in strength and remained in the air for some time after they themselves were silent. While the outdoor free field was excellent for the communication of precise semantic information (e.g., spoken language), the diffuse field of the cave must have seemed ethereal and otherworldly for those early humans entering it for the first time. It was not ideal for semantic communication, but the aesthetic of the space itself became useful for other forms of communication, such as music or religious rituals that benefited from the mystery added by long reverberation times which were rarely experienced by their listeners. Figure 2 shows a basic continuum, ranging from mid-frequency reverberation time values very near zero to those that are much larger [the highest mid-frequency *T* values measured in physical churches are about 12 s Girón et al., 2017].

It is a crucial problem in acoustical design that subjective descriptors associated with both ends of the continuum are rated as preferable by audiences, and yet these parameters are basically in complete opposition to one another (Schroeder et al., 1974; Rasch and Plomp, 1982; Kosała and Engel, 2013). “Clarity,” “directness,” and “intelligibility” are associated with the free field, while “envelopment,” “immersion,” and “spaciousness” are associated with the diffuse field, yet both of these extremes cannot exist perfectly in the same physical room. Because of this, it is difficult to speak of “good acoustics” as a single ideal, as any particular room must involve a compromise of opposing values based on the intended use of sound in that space. Mid-frequency reverberation times will be reported only for historically significant examples in this article; for a more thorough catalog of reverberation times in European churches the reader is referred to Giron's extensive review in Girón et al. (2017).

However it should be emphasized that other qualitative changes could move an auditory experience to the left or right on this continuum independent of reverberation time, although reverberation is highly correlated with it: for instance, if a piano piece is played in a dry church without the sustain pedal, and then the sustain pedal is applied, the overall sound experience has shifted toward the Cave away from the Open Air, even though the reverberation of the room itself is unaltered. If a musician turns their back on their audience, the direct sound will be attenuated, and the overall auditory experience will become more diffuse. In this case the listener perceives the space as though it had a greater *T* value, though the objective value has not changed (since that parameter is defined based on an omnidirectional sound source). Non-auditory factors, such as visual components, may also influence the subjective experience of a congregant in attendance during a service (Jeon et al., 2014). In the case of liturgy, the position or orientation of a priest, whether the priest is speaking or singing, and the language in which the service is conducted can all affect the liturgical space's clarity, independent of the physical acoustic clarity in the architectural space alone.

### 2.2. Religious History

Having stated this, one may wonder what it has to do, if anything, with religion. It does not require too much imagination to see an analogous tension within many different fields, generally categorized as “semantic” on one end and “aesthetic” on the other. Semantic forms privilege the conveyance of clear information through a channel, while aesthetic forms seek enjoyment through the embodied experience that arises from the channel itself. The opposing acoustical poles of Semantic and Aesthetic described here also mirror a related dichotomy observed in the Cognitive Science of Religion (CSR). CSR scholar Harvey Whitehouse's “modes of religiosity” theory groups religious movements into the *Imagistic* and *Doctrinal*. In Imagistic modes of religiosity, “the transmission of central theological insights is through rarely performed but highly emotional events” (Barrett, 2007). The opposing Doctrinal mode of religiosity focuses on explicit teaching and “low-arousal theological transmission events.”

Anyone who has heard music performed in a large stone church can attest that such an environment is not ideal for the conveyance of exact lyrical phrasing, nor even perhaps for the melody of the music being performed. Yet there is an aesthetic, “other-worldly” quality that goes along with such an environment that lends support to its worldview of transcendence of some sort. This general tension between semantic and aesthetic content remains: the religion still wants to be able to convey the Word (the normative principles of the faith) but also to convey the Mystery (the existential experience of “the life of the world to come”). In Whitehouse's paradigm, high-reverberation spaces are better suited for the Imagistic mode, while acoustically dry spaces are better suited for the Doctrinal mode.

Most of the examples presented here will be focused on Western music, history, and the Judeo-Christian tradition. This is partly because of the author's own familiarity with the subject matter. In addition, since modern room acoustics originated within the West, we have much more data characterizing Western performance halls and worship spaces than for other traditions. This is beginning to change, however, as new studies have begun to investigate the role of acoustics in worship spaces within Islam (Hammad, 1990; Abdelazeez et al., 1991; Karabiber, 2000; Abdou, 2003; Kleiner et al., 2010a; Ismail, 2013), Hinduism (Prasad and Rajavel, 2013), and Buddhism (Soeta et al., 2013; Jeon et al., 2014). In each case, something like the semantic–aesthetic tension arises, showing that these acoustical categories are not in any way exclusive to the West or Judeo-Christian faith traditions.

## 3. Christianity and Acoustics

The history of Western performance spaces is not rooted purely in religious use, but rather, in the “open-air” case, in the Greek amphitheaters (Chourmouziadou and Kang, 2008; Mo and Wang, 2013; Bo et al., 2018), and in the reverberant case, in the Greek *odea* or music halls (Farnetani et al., 2008; Rindel, 2011). However, these prove to be the exception rather than the rule, as for over a thousand years Western performance spaces were predominately religious in function. This would create a strong link between political and theological concerns and the spaces in which new works of music would be composed and performed.

The early Christian church began more or less as a splinter sect of Judaism. For this reason, early Christian liturgy was centered around the practice of Judaism, but not of the Jewish temple, which had been destroyed in 70 AD. While the Temple was a larger space whose worship included instruments and sung psalms (Kleiner et al., 2010b), the Jewish synagogue was smaller, less reverberant, and focused on the spoken word rather than music (McKinnon, 1979). The early Christian faith faced disapproval or outright persecution from the Roman Empire during its first few centuries, and as a result its worship was primarily confined to house churches or *domus ecclesiae*, small spaces in which a primarily semantic spoken liturgy was acoustically appropriate. These *domus ecclesiae* in general had very low reverberation times and high acoustical clarity compared to the later *domus Dei*, larger buildings intended to serve primarily as worship spaces (Suárez et al., 2013). The New Testament contains many references to early Christian singing, but these were probably derived from private Jewish family worship rather than the larger-scale worship ceremonies of the synagogue (Smith, 1984).

Constantine I issued a policy of toleration and legalization of Christianity in the early 4th century, and soon after Christian churches moved into newly built structures, modeled off of Roman *basilicas*, which were large enclosed public meeting spaces. Some of these spaces had reverberation times to the far right of our continuum: one of the largest basilicas built during this period, San Paolo fuori le Mura in Rome (consecrated 324) has a mid-frequency reverberation time over 9 s, and the other three primary Roman basilicas possess values from 5 to 7 s (Shankland and Shankland, 1971). The dramatic political swing resulting from Constantine's conversion also led to a dramatic acoustical swing from the very “dead” acoustics of a house church to the cave-like acoustics of the new basilicas. The emergence of a codified “gradual” sung psalm during the Christian liturgy seems to also have arisen during this same period (McKinnon, 1987). Acoustician David Lubman has made an extended argument that this change from a spoken to a primarily sung liturgy was in reaction to the time-dispersive characteristics of the new basilicas (Lubman and Kiser, 2001).

**Figure 3:**
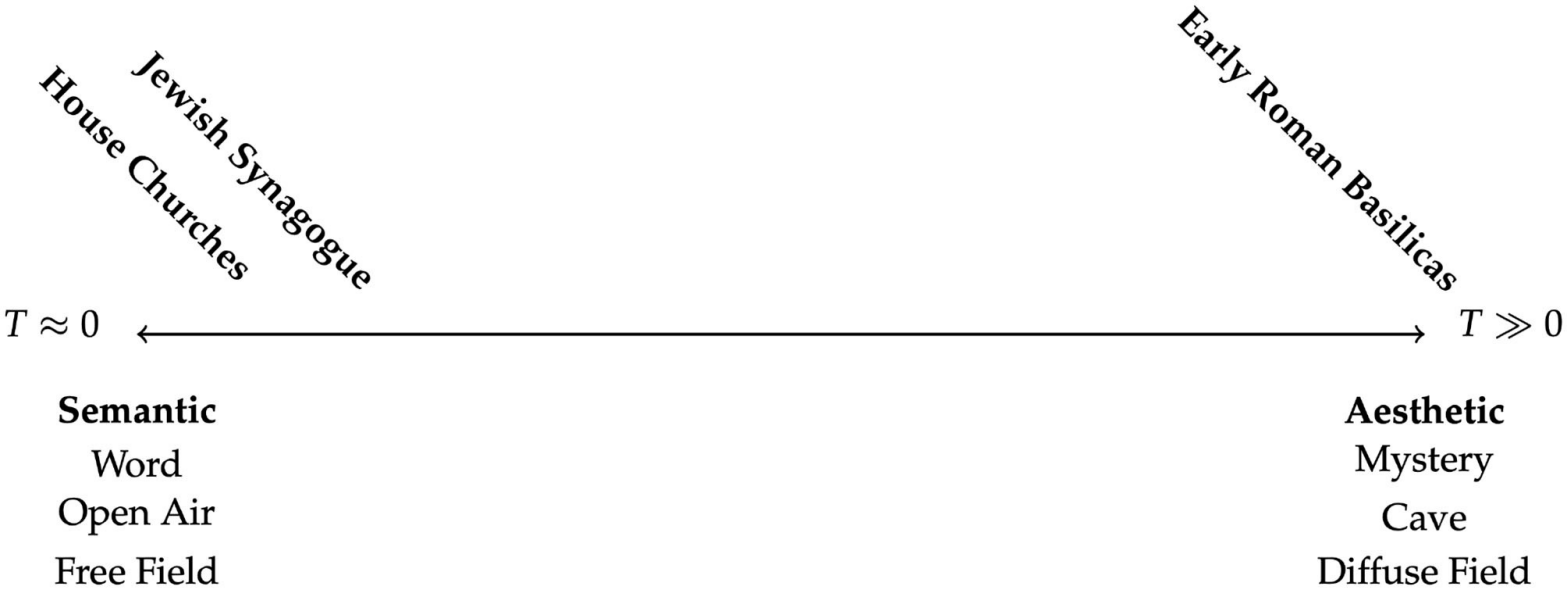
Continuum of Liturgical space, circa 500.

If we were to plot these three worship styles—Jewish synagogue, Christian house church, and early Christian basilica—on the continuum of liturgical space it might look something like that shown in Figure 3. The extreme shift in acoustic context causes a shift in liturgy, from spoken words to a musical liturgy that is more appropriate for conveying aesthetic experience and emotion than precise semantic meaning.

The proto-Romanesque churches that followed the early basilicas changed in some ways, but maintained large stone enclosures, ensuring very long reverberation times. Over the next 1000 years, these reverberant spaces, along with the codification in the 8th century of a series of monophonic plainsong chant melodies which would become known as Gregorian Chant, gave rise to a set of circumstances in which congregants throughout Christendom heard not only the same sequence of chanted notes, but also a similar sustaining effect, similar to holding down the sustain pedal long before the modern piano was invented. Jaime Navarro argues that

The greater reverberation time…[made] the listener simultaneously hear notes different to the unison. This not only conditioned melodic cadence, but over time it also ensured a certain education and familiarization with polyphonic sound. Indeed, the persistence of the sound of the different notes of the melody leads to melodic developments, structured in such ways that the simultaneity of notes is resolved over certain harmonies. As a result of this, the location (the medieval church) is the main reason why music followed a given direction (polyphony). Acoustics had an influence on the development of music (Navarro et al., 2009).

This acoustical theory of the origins of Western polyphony is not a mainstream conclusion among musicologists, but is (perhaps less surprisingly) often accepted by acousticians (Lubman and Kiser, 2001; Boren, 2017), often focusing on the work of the Notre Dame School of Paris in the 12th and 13th centuries. If it is to be believed, however, it provides a strong link between acoustics and the various religious disputes over polyphony that would occur within the Catholic and later Protestant churches as well. The slow, monophonic chants practiced in reverberant spaces would sound perhaps too clear and even redundant if sung within a dry, small volume. Yet in the right setting this type of chant, with its redundancy and extra clarity, can flourish due to the diffusive nature of large stone churches to complicate the acoustic signal introduced into them. At any rate, polyphonic compositions became normal for festive occasions by around 1400, and by 1450 were becoming common in settings of the ordinary Mass as well (Bossy, 1985).

In addition to the musical development proceeding within the church, architectural styles also created acoustical changes in the soundscapes presented during the mass. The Romanesque churches had maintained long reverberation times but also increased in complexity, adding decorative ornamentation that had the effect of increasing acoustic diffusion and spreading sound more evenly throughout the space. The Gothic churches that followed them increased in height and total volume while still predominantly using stone and other reflective materials in their construction, yielding increased reverberation times and reduced diffusion (Lubman and Kiser, 2001). While there are nearly always tradeoffs with regard to the length of a space's reverberation time, the addition of more diffusion, or uniform scattering of sound throughout a space, is nearly always desirable, and its absence can lead to acoustic effects in the time domain (e.g., flutter echo) or frequency domain (e.g., modal coloration) that are usually perceived as detrimental (Rubak, 2004).

It should be noted that from about 850–1054 the Eastern churches were gradually pulling away from the Western Roman Catholic church, partly over issues of theological doctrine, and partly over the question of papal supremacy, i.e., whether the Bishop of Rome (the Pope) had authority over other bishops in the East. Though Eastern Orthodoxy is not the primary focus of this article, its acoustics, liturgy, and theology are also linked. While Roman Catholic canon law “came more and more to see theology as a kind of philosophy,” Eastern Orthodoxy conversely resisted “rationalistic systematization” and emphasized mystery, mysticism, and paradox in its theology (Olsen, 1999; Placher and Nelson, 2013). The faith is held together not by the intellectual resolution of this mystery, but by the practice of its liturgy, as encompassed in its rule *Lex Orandi, Lex Credendi* (“the law of worship is the law of belief”). Meanwhile the worship and liturgy of Eastern Orthodoxy was uniquely shaped by the practices of its “Great Church”—the Hagia Sophia in present-day Istanbul. The Hagia Sophia's reverberation time has changed over history with different configurations (Rindel, 2002), but in its current empty state the value is about 11 s (Abel et al., 2013), emphasizing “mystery” over “word” perhaps even moreso than the early Roman basilicas. In addition, the Hagia Sophia's large, stone Greek cross layout would become a blueprint for other churches in the Orthodox tradition (Małecki et al., 2017).

## 4. 16th Century Reformation Movements

The Protestant Reformation in the West is often subdivided into the Lutheran, Calvinist, and Anglican Reformations, taking place in Germany, Switzerland, and Great Britain, respectively, as well as the Radical Reformation or Anabaptists in parts of both Germany and Switzerland. The Lutherans would spread north into Scandinavia, while Calvinism also spread into England, Scotland, and the Netherlands. The Radical Reformers, opposed by other Protestants as well as Catholics, mostly died out except in particular pockets, surviving today in Mennonite and Amish communities (Placher and Nelson, 2013).

Some larger historical trends during the 16th century may have contributed to the Reformation's rapid onset, including rapid population growth and the influx of precious metals from the New World, both of which contributed to significant monetary inflation. The economic pressure exerted by these factors led to increased urbanization and a stronger merchant culture in some countries, while others reacted instead by shoring up the older system of peasant serfs farming on larger manor houses. In general the former countries were home to expanding literacy and the expansion of universities, and they were more likely to become hotbeds of the Reformation. Rising literacy rates had also led to the growth of preachers who delivered written sermons rather than simply reciting the Mass, and these preachers were disproportionately likely to join the Reformation. In contrast, countries with greater reliance on agricultural estates were more likely remain Catholic (Spitz, 1985). Thus, the demographics most involved in the Reformation had become accustomed to direct access to knowledge without intermediaries, a preference that would manifest itself in their churches and liturgy.

### 4.1. Lutheran Churches

“I have only put God's Word in motion through preaching and writing. The Word has done everything and carried everything before it.” –Martin Luther, 1522 (Spitz, 1985)

#### 4.1.1. Vernacular Language

While these intellectual developments were taking place, it might be useful to consider the perspective of the average parishioner attending mass in a medieval church. In contrast to a house church around 300 AD, which would have featured a spoken word liturgy in a small space to people who generally all spoke Latin, 1000 years later a churchgoer would have experienced a sung liturgy in a highly reverberant space, in a language he or she likely did not speak. In addition, since many in the congregation were also visually unable to see the priest or choir, this disorienting combination of a foreign language and high reverberation time were the primary experience for most of the laity. While acoustics may not have been among the most important explicit factors involved in the Protestant Reformation, it is arguably aligned with many of the causes of the split.

The existing ecclesiastical hierarchy presented a faith that was altogether mysterious to the average churchgoer, and somewhat incomprehensible along various lines. It stressed a “blinder” sort of faith in the decrees of the *magisterium*, whose authority was conveyed through both the language and the soundscape of the mass, which was wholly mysterious to many of the attendees. In contrast, the pre-Reformation trend from John Wycliffe in England to Jan Hus in Bohemia stressed the ability of the individual to know spiritual reality through direct contact with scripture, leading to repeated calls for vernacular translations of the Bible. This trend intensified in the later Reformation theology of Luther and Calvin as well as humanists like Erasmus, all of whom stressed the importance of vernacular language and the individual's comprehension of the language used during worship. Luther himself rewrote the traditional order of worship to give the vernacular sermon a more central position (Spitz, 1985) and later claimed at the dedication of a new church that “nothing else should take place in it other than our dear Lord will speak to us Himself through His Holy Word and we, in our turn, speak to Him through prayers and songs of praise” (Petzoldt, 1999).

The 16th century Reformation writ large can be seen acoustically as a long-awaited counter swing back toward the semantic. As the reformers took over Catholic churches and built their own new worship spaces, the acoustics of these churches followed the presuppositions of the movement. The focus of the worship service was no longer the intermediation of the priest and the dispensation of grace through the church. The Reformation focus on the “priesthood of all believers” gave priority to the worship service as a gathering of individuals who were each granted direct access to God via scripture, and thus the focus of the gathering shifted to emphasize the proclamation of the Word rather than only the Mystery of the communal church itself.

#### 4.1.2. Lutheran Alterations

As the Lutherans took over many Catholic churches throughout Germany, they made some alterations primarily for theological reasons, while others may have been influenced by acoustic considerations. In addition to the selling of papal indulgences, perhaps the primary catalyst for the Reformation, many churches also contained side altars and chapels and received additional funds for holding special masses for families or confraternities. To the Lutherans this smacked of selling salvation at the institutional level and idolatry at the level of the individual. This led to many ensuing rounds of iconoclasm initiated by Andreas Bodenstein von Karlstadt and others wherein side altars, statuary, and icons were removed or destroyed (Spitz, 1985). Luther himself was more moderate on this issue (and many others), and he emphasized that good art, like good works, had value but did not have salvific properties (Luther, 2003). As long as such images were not worshiped, Luther considered them *adiaphora* meaning that they were optional but did not need to be removed (Heal, 2011). The result for the visual arts, known as didacticism because it emphasized art's role in teaching about the Word, “required that the image became less rather than more: less visually seductive, less emotionally charged,” so that its content could be delivered smoothly without arousing the passions of its viewers (Koerner, 2003). A more severe iconoclasm and didacticism would be enacted in Calvinist areas where Luther's moderating caution was not present, whereas a large portion of the Catholic ornamentation would remain in Lutheran churches after the Reformation (Heal, 2011).

The side altars, chapels, baptismal fonts, images, and statues that many Reformers viewed as idolatrous served an important acoustic role in these reverberant churches by scattering reflections off parallel walls and ensuring good sound diffusion throughout these spaces, avoiding flutter echoes or severe modal coloration and helping to equalize the reverberation time throughout the space (Meyer, 2009). High amounts of diffusion increase signal decorrelation at a listener's ears, subjectively increasing ratings of sound immersion and envelopment (Kendall, 1995), which are qualities obviously related to the “Diffuse Field” side of the continuum considered here. The acoustic effects were incidental to the intentions of both those who created them and those who destroyed them. Yet they had salient consequences nonetheless and more so by their absence in the more extreme Calvinist worship spaces that would follow.

Most Christian churches at this time had a main altar against the east wall of the chancel, and during the Mass the priest would turn his back on the congregation in the posture known as *ad orientum*, appealing to God on behalf of the congregation. Luther disagreed with this, and argued instead that the altar should be in the center of the chancel (Lund, 2002), allowing the priest to face both the altar and the congregation (*versus populum*), which would have increased speech intelligibility significantly in smaller churches due to the sound radiation pattern of the human voice. Luther's suggestion was not widely adopted in his lifetime, though philosophically it is in keeping with many of the tenets of his movement.

In place of the removed side chapels and altars, however, the Lutherans built extra galleries and ensured clear sight lines from new seats to the pulpit, elevating the importance of the spoken word during the service. The clear sight lines increased the strength of direct sound, while the corresponding increase in absorption (probably accidental) from the additional galleries also reduced the reverberation time significantly in many cases, while also increasing intelligibility. Charles Sanford Terry summarizes this change: “The congregation no longer assembled to adore a Mystery accomplished out of sight and hearing, but to listen to the homily of a preacher elevated and visible” (Terry, 1931).

**Figure 4:**
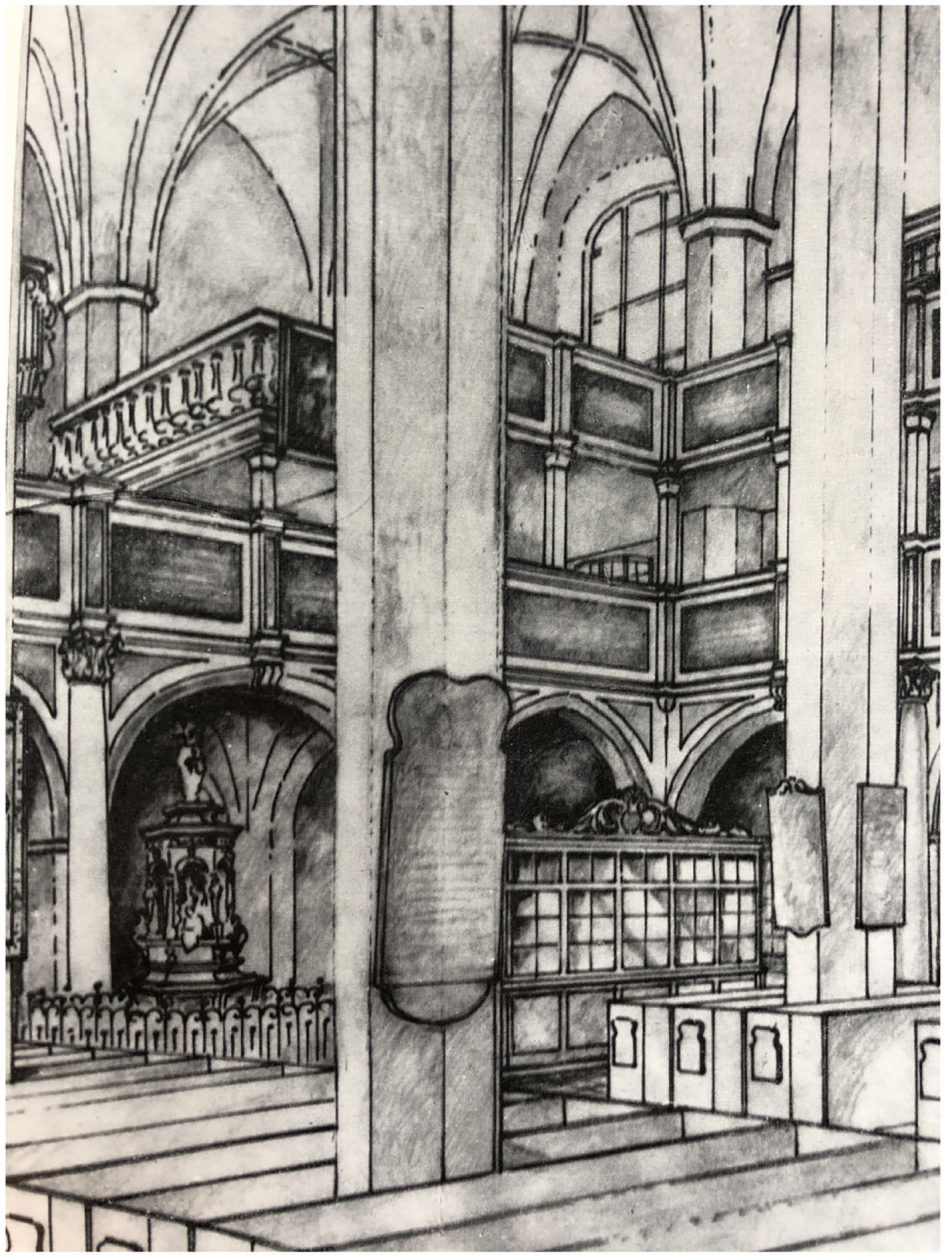
Drawing of Lutheran double galleries in 16th century, Thomaskirche, Leipzig, Germany (Stiehl, 1984).

The Thomaskirche in Leipzig would undergo many alterations after the Reformers took control of the city, though many of these were primarily visually motivated (Petzoldt, 2000; Boren et al., 2019). Figure 4 shows a drawing of the double galleries added to the Thomaskirche in Leipzig as they would have existed during the 16th century (the upper gallery no longer exists). Hope Bagenal surmised that the ensuing increase in acoustic clarity in Leipzig's Thomaskirche provided the necessary acoustic environment for J.S. Bach's musical innovations there in the 1700s, especially his use of dramatic tempo modulations (Bagenal, 1951). C.P.E. Bach also claimed that his father was very sensitive to the acoustics of any space he visited (David et al., 1998), and thus the Lutheran alterations to the Thomaskirche might reasonably be assumed to have had an effect on the music he composed for that space. Bach's relation to Lutheran theology more specifically is covered in detail in Leaver (2000).

Overall, it must be remembered that Luther ushered in many changes that provided a counter-swing back toward the clarity of the spoken Word, but he was in many respects a moderate compared to later reformers. He maintained some vestige of a priesthood, and, perhaps because of his training as a musician, he also kept parts of the Latin Mass and adored the polyphony of Josquin particularly (Bossy, 1985). Perhaps because Luther's own life was saved by the actions of Frederick the Wise, Elector of Saxony, after Luther's excommunication from the Catholic church, Luther was more positively disposed toward royal authority and tended to call for less radical political change as a result of his ecclesiastical reforms. The reforms undertaken by Calvinists in Switzerland and parts of Germany tended to be more extreme, both politically and architecturally (Heal, 2011).

### 4.2. Calvinist Churches

“For by his Word, God rendered faith unambiguous forever, a faith that should be superior to all opinion.” –John Calvin, *Institutes* (Calvin, 2006)

Though the Lutheran churches represent a significant turn away from the acoustics of pre-Reformation Catholicism, the churches built in the Reformed or Calvinist tradition were more extreme, both in theology and in their architectural rendering of that theology. Whereas, Luther tended to accept architectural or musical innovations if they were not expressly forbidden by scripture, the Swiss Reformation, beginning with Huldrych Zwingli (1484–1531), tended to only sanction forms of worship that were expressly *authorized* by scripture, leading him to strip statues, images, and even pipe organs from churches in Zurich (Spitz, 1985).

Zwingli's successor as leader of the Swiss Reformation was John Calvin (1509–1564). Calvin left France to pursue the life of a scholar, but ended up in charge of Reformation Geneva in Switzerland, a city that had obtained self-rule in the 14th century (Placher and Nelson, 2013). Calvin continued to pursue scholarship in the form of numerous writings on Christianity and theology while ushering in many reforms to the church and state in Geneva and beyond. Though he had Platonic tendencies with respect to abstract and universal ideals, Calvin went a step farther than Plato, who claimed that humans sin because they are ignorant; in contrast, Calvin insisted, we are ignorant because we sin. This stress on the inability of humanity to see clearly apart from God's Word led Calvin to de-emphasize sensory knowledge, including the aesthetic input of the senses during a worship service, preferring instead the semantic content of the preaching of the Word only. In his *Institutes*, commenting on the Second Commandment (“You shall not make yourself a graven image…”) Calvin states that the commandment “withdraws us from petty carnal observances, which our stupid minds, crassly conceiving of God, are wont to devise,” and that worshippers must not “subject God, who is incomprehensible, to our sense perceptions, or …represent him by any form” (Calvin, 2006).

This focus led to a greater tendency for iconoclasm than among the Lutherans, to the extent that many Lutherans considered themselves to be charting a middle path between “iconoclastic Calvinists and idolatrous Catholics” (Heal, 2011). The Calvinist meetinghouses represented a stripping away of all the ornamentation that was seen as nothing but vestigial dead-weight from years of Catholic heresy. The extreme change in architectural style led to the meetinghouses being called “four walls and a sermon” (Rath, 2003) or more derisively, resembling “a public beer hall rather than the temple of the Lord” (Heal, 2011). These spaces were generally built as simple one-story volumes, only enlarged when the congregation had grown enough to require balcony seating (Rath, 2003). This had the effect of dramatically reducing reverberation time, leading to spaces appropriate for the spoken word but offering poor support for almost any form of music.

During the reign of the Catholic Mary I (“Bloody Mary”) in England (1553–1558), many English Protestants fled to Switzerland and were strongly influenced by the Calvinist tradition. Many of these refugees later returned to the British Isles and became known as the Puritans, who exerted a strong influence on the English and Scottish Reformations. They implemented architectural reforms, such as the destruction of rood screens and side altars, and whitewashed the walls of churches (Spitz, 1985). The Puritans would also go on to build many of the earliest houses of worship in the American colonies. These meetinghouses tended to be rectangular or even square, with simple wooden walls. In addition, the stripping of this ornamentation had the unintentional acoustic consequence of greatly reducing diffusion, leading to modal resonances and flutter echoes throughout the churches that would have been detrimental to virtually any use of the space, whether semantic or aesthetic. This style persisted in the early Puritan meetinghouses of New England as well (Rath, 2003). A similar though less severe aesthetic can be found in the 51 Anglican churches Christopher Wren designed for London after the Great Fire of 1666, which the architect specified should be small enough that the entire congregation could both see and hear the rector clearly (Navarro et al., 2009). Later Calvinist preachers, such as George Whitefield would go further and preach mainly outdoors, an acoustic environment with great clarity but almost no sound reflection, the ultimate distillation of the Calvinist focus on speech intelligibility (Boren, 2016). Overall the Calvinist worship spaces can be thought of as the most extreme counter-swing back to the highly semantic house-churches of early Christianity: small, good for the spoken word, and not well-suited for music or mystery.

### 4.3. Catholic Churches

Meanwhile, in Catholic Europe the series of reforms known as the Counter Reformation also manifested in ideas about intelligibility and clarity. Perhaps because of the presence of the papacy, Italy never contained a critical mass of Reformers, such as existed in Germany or Switzerland. But throughout the 1530s Italy was home to a Catholic “evangelism” movement which stressed the value of scripture and the spoken Word. Some of this movement's members eventually became Protestant, while others remained to pursue reform within the Catholic Church itself (Spitz, 1985).

Though the story of Palestrina singlehandedly saving polyphony through his Pope Marcellus Mass is mostly a myth, it is true both that the Council of Trent (1545–1563) considered musical intelligibility an important issue, and that Palestrina himself was an advocate of greater clarity in musical composition (Monson, 2002). Like Luther, the Council still affirmed the use of architecture, art, and ornament to convey Catholic theology, and as a result, the Baroque churches that followed showed somewhat reduced reverberation times, while still maintaining excellent diffusion through extensive physical ornamentation (Navarro et al., 2009). A draft of proposed Mass reform (though not officially adopted by the Council) from 1562 illustrates well the Counter-Reformation's attention to both Word and Mystery (emphasis added):

Canon 8. Since the sacred **mysteries** should be celebrated with utmost reverence, with both deepest feeling toward God alone, and with external worship that is truly suitable and becoming, so that others may be filled with devotion and called to religion: …Everything should indeed be regulated so that the Masses, whether they be celebrated with the plain voice or in song, with everything **clearly and quickly executed, may reach the ears of the hearers and quietly penetrate their hearts** (Monson, 2002).

Some Italian Catholic churches which possess high reverberation times today while empty were significantly less reverberant on festive occasions (for which Masses were chiefly composed) because of decorative tapestries and large crowds (Hopkins, 2006; Martellotta et al., 2008). However, the lack of support from reflected sound meant that the choir's overall loudness would decrease as well. Thus, for a listener near the nobility at the front of the nave, the effect of a polyphonic choral performance would be much better than for the present day empty church. Unless the crowds themselves were completely silent (unlikely for large gatherings), the priest's spoken words would likely be obscured for most attendees in the nave (Boren et al., 2013).

In Italy the greater similarity between spoken Italian and Latin meant that the spoken words of the Mass were more intelligible to the average parishioner without the need of translation into the vernacular as in other European countries. In the church of San Francesco della Vigna in Venice, the humanist friar Francesco Zorzi wrote a document in 1535, a decade prior to the Council of Trent, advocating for a coffered wooden ceiling, because he believed it would improve the intelligibility of the sermons spoken from the pulpit. However, his proposed ceiling was not built for cost reasons, and the church was given a plaster vaulted ceiling instead (Howard and Moretti, 2010). A computer simulation of the ceiling Zorzi proposed showed that the speech intelligibility in the space would not have been significantly improved by his alteration, because the ceiling was too high to produce strong early reflections or reduce reverberation time (Boren and Longair, 2012; Bonsi et al., 2013). Thus, even when intelligibility was an explicit goal of a new construction, the culture's understanding of acoustics was sometimes insufficient to attain it.

One significant Catholic church known to have been altered during the Counter Reformation is the Cathedral of Amalfi, which dates back to the sixth century. The original single nave was expanded with two additional naves in the 10th century, but during the Counter Reformation this was reduced back to the single nave structure it retains today (Museo Diocesano, 2018). Though the stone composition of the space was still reflective, the smaller volume reduced reverberation and increased speech intelligibility, albeit for a smaller congregation.

**Figure 5:**
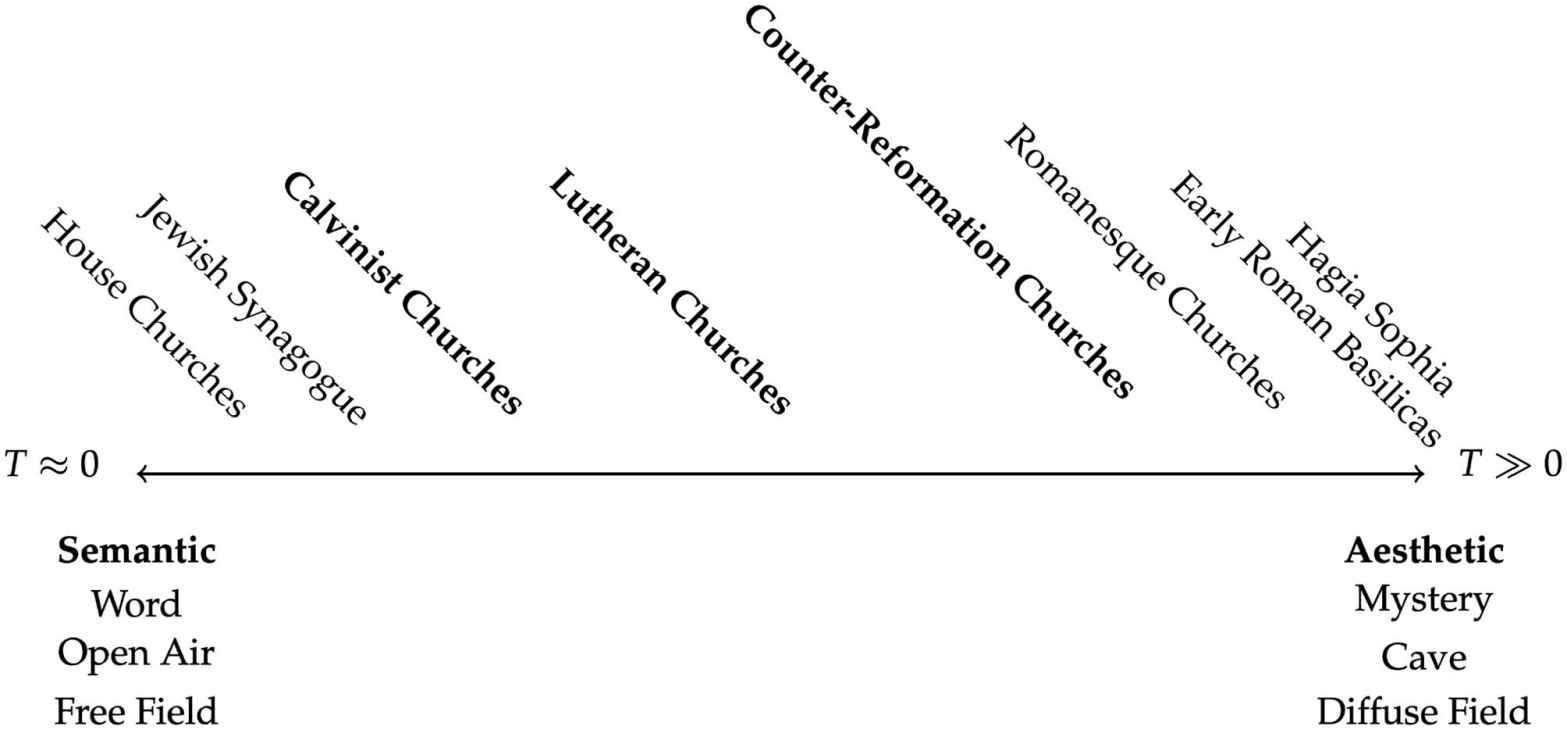
Continuum of Liturgical space, circa 1700.

The Counter Reformation's focus on greater clarity could be seen as a slight move away from “Mystery,” though much less so than in the case of the Calvinist or even Lutheran examples. An update of the acoustical continuum by the year 1700 (Figure 5) might see these three traditions—Calvinist, Lutheran, and Catholic Counter-Reformation, occupying space toward the center, though each still possesses doctrinal positions that prize the free field or diffuse field.

## 5. Discussion

### 5.1. Correlation or Causality?

Winston Churchill, when commenting on the importance of layout of the House of Commons to British democracy, famously said “We shape our buildings; thereafter they shape us” (Bremner, 2016). This dynamic can be seen in secular and public spaces, but equally so in worship spaces: the medium is shaped to fit the message, but thereafter the message itself is “locked in” to the medium in which it propagates. A 16th century priest in a highly reverberant cathedral might want to spend more time on his sermon, but he would be physically hindered by the acoustic channel in which his words echoed. It would have been equally hard for a musically-minded Calvinist minister to introduce a choir, as the meetinghouse's acoustics ensured that such music would sound tinny and hollow.

This essay has focused primarily on showing the correlations between theological focus and acoustical clarity, but the question of causality is much more complex: does theology necessarily lead to a change in acoustics, or vice-versa? In many of the cases, theological principles led to architectural changes without a holistic understanding of the acoustical ramifications, which themselves affected the liturgy within the same space. In the long run, changes to the liturgy can likewise implicitly shape theology, leading to a possible feedback cycle in which the differences between each tradition become greater over time.

But in other cases the chain of causality did not necessarily begin with theology, such as Constantine's policy of toleration, which was primarily a political change. Similarly, George Whitefield preached outdoors not because of his own theological preference but because his evangelical message had gotten him banned from most of the establishment Anglican churches. Only in the most basic application of acoustics—the direct sight line to the preacher—did the Lutherans arguably make a change primarily for acoustic reasons. In the rare example where we have documented evidence of attention to acoustics, such as Zorzi's letter, economic considerations eventually bore out over his acoustical argument. For many churches we still do not have enough documentary evidence about the acoustical and liturgical evolutions to be sure of the chain of causality at every step.

### 5.2. More Recent History

Of course there has been much movement on the continuum of liturgical space since 1700, but within architectural acoustics most of the innovation since that time began to be driven by the construction of secular music halls rather than religious architecture (Forsyth, 1985). However, some recent trends have affected the Word/Mystery balance in both practice and architecture of worship services and spaces. In the 20th century, many Lutherans did adopt Luther's suggestion of the priest facing *versus populum* (Lund, 2002), but also most Catholics adopted it through the resolution of The Second Vatican Council (1962–1965). In addition, many Catholic churches abandoned the Latin mass after the Council, surely a significant liturgical push toward the semantic and away from the aesthetic (Navarro et al., 2009).

**Figure 6:**
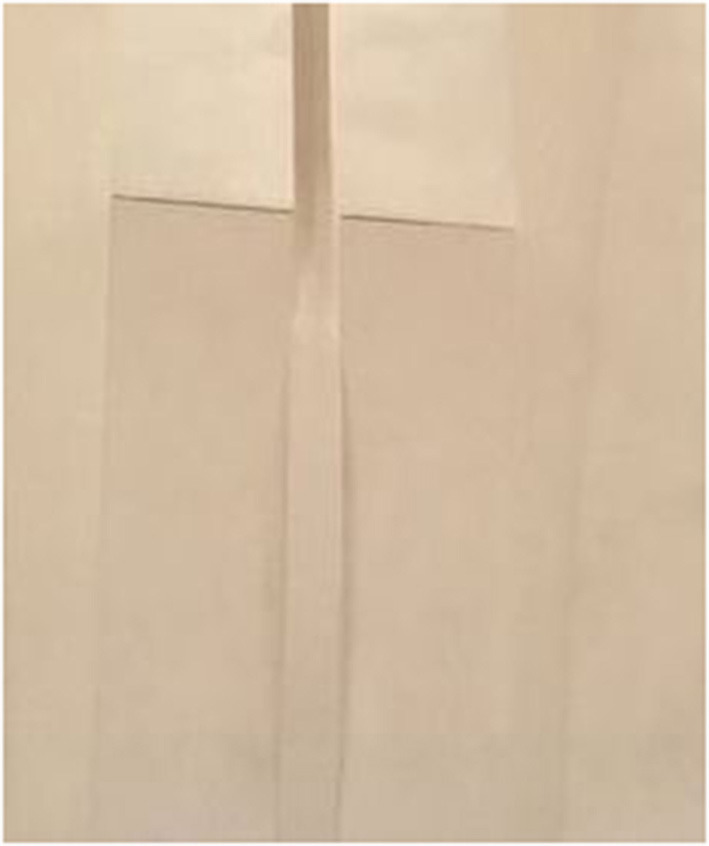
Bare-yet-diffusive wall design in the W. 83rd Ministry Center.

A newer example of reformation in acoustic design is the West 83rd Ministry Center, built by Redeemer Presbyterian Church on the Upper West Side of Manhattan in 2012. This was the first new church constructed in Manhattan in over 30 years, designed by Gertler & Wente, with acoustic design by Harvey Marshall Berling Associates. Redeemer as a Presbyterian institution stands firmly in the “Word” category of Calvinist doctrine, its services each week featuring a 30-min sermon on the day's scriptural text. The space reflects this stance, possessing a reverberation time slightly over 1 s when filled (based on data obtained from the designers) and eschewing elaborate ornamentation. Despite the plain walls reminiscent of the English Puritans' whitewash, the walls in this space are purposely offset at small angles to increase diffusion, which is also improved through an innovative depth-carved cross behind the main stage (Figure 6). This reduces the acoustical problems prevalent in the earlier, rectangular meetinghouses and provides a more even distribution of reflections for musical performance.

Even within older churches that still possess large reverberation times, the advent of distributed loudspeakers throughout the space can increase the direct speech of a preacher enough to ensure intelligibility even in the midst of long decay times, another significant step toward overall clarity. This technique was pioneered in St. Paul's Cathedral in London, whose mid-frequency reverberation time was measured at 11 s when empty (Girón et al., 2017). Rather than attempting to introduce large amounts of absorption into the space, it was decided to use electroacoustic reinforcement to increase the strength of the direct sound relative to the late reverberation, ensuring adequate speech intelligibility (Parkin and Taylor, 1952). Other more absorbent church constructions conversely possess low reverberation times but can add digital reverberation to individual singers or instruments, adding Mystery when desired and removing it when greater clarity is required (e.g., during the sermon).

Some older churches whose altars prevent the priest from facing the congregation still use the *Ad Orientum* posture, as in the Thomaskirche in Leipzig. In this space, when the priest is near the church's crossing, she is unamplified and faces the congregation. However, when facing the altar in the chancel the priest speaks into a microphone and is heard through a distributed set of loudspeakers located on the columns in the nave. In this case electroacoustic reinforcement allows the liturgical space to retain the visual percept of a priest advocating on behalf of the congregation, while the auditory percept is kept sufficiently clear so that the semantic content of that appeal is still heard and understood by those in the nave.

## 6. Conclusions

Along the continuum of liturgical space constructed here, there is a long history within the Judeo-Christian tradition to oscillate between the semantic and aesthetic poles. These oscillations may be caused by chance circumstance (as in the move into large basilicas in the 4th century) or by explicit theological reaction (as in the case of the Reformation). As the church divided, individual traditions' explicit emphases were reflected in the liturgical spaces they inhabited: crisp clarity of the Word in the case of the Calvinists, a much greater emphasis on Mystery in the Catholic Counter-Reformation, and a moderate state of compromise in the case of the Lutherans.

To hazard an acoustical metaphor, the historical system is something like a dampened oscillator, as these oscillations have decreased in magnitude over time. The various traditions are converging from different directions, somewhere near where the Lutheran churches ended up, in the middle. The Catholic churches are becoming more clear, and the Calvinist churches are becoming more diffuse. The application of electronic reinforcement has made it possible to change the overall liturgical space significantly during the course of a single service, allowing even more room for compromise. Despite the various doctrinal issues separating the many denominations, in the auditory domain at least, the trend seems to be toward greater future unity.

## Author Contributions

The author confirms being the sole contributor of this work and has approved it for publication.

## Conflict of Interest

The author declares that the research was conducted in the absence of any commercial or financial relationships that could be construed as a potential conflict of interest.
